# Cultural traits operating in senders are driving forces of cultural evolution

**DOI:** 10.1098/rspb.2023.2110

**Published:** 2024-03-13

**Authors:** Magnus Enquist, Fredrik Jansson, Stefano Ghirlanda, Jérôme Michaud

**Affiliations:** ^1^ Centre for Cultural Evolution, Department of Psychology, Stockholm University, Stockholm, SE–106 91, Sweden; ^2^ Department of Zoology, Stockholm University, SE–106 91 Stockholm, Sweden; ^3^ Division of Mathematics and Physics, Mälardalen University, SE–721 23 Västerås, Sweden; ^4^ Institute for Futures Studies, SE–101 31 Stockholm, Sweden; ^5^ Department of Psychology, Brooklyn College, 2900 Bedford Avenue, Brooklyn, NY 11210, USA; ^6^ Department of Psychology, CUNY Graduate Center, 365 5th Avenue, New York, NY 10016, USA

**Keywords:** cultural evolution, cultural transmission, cumulative culture, dynamical systems, trait-individual duality, developmental psychology

## Abstract

We introduce a mathematical model of cultural evolution to study cultural traits that shape how individuals exchange information. Current theory focuses on traits that influence the reception of information (receiver traits), such as evaluating whether information represents the majority or stems from a trusted source. Our model shifts the focus from the receiver to the sender of cultural information and emphasizes the role of sender traits, such as communicability or persuasiveness. Here, we show that sender traits are probably a stronger driving force in cultural evolution than receiver traits. While receiver traits evolve to curb cultural transmission, sender traits can amplify it and fuel the self-organization of systems of mutually supporting cultural traits, including traits that cannot be maintained on their own. Such systems can reach arbitrary complexity, potentially explaining uniquely human practical and mental skills, goals, knowledge and creativity, independent of innate factors. Our model incorporates social and individual learning throughout the lifespan, thus connecting cultural evolutionary theory with developmental psychology. This approach provides fresh insights into the trait-individual duality, that is, how cultural transmission of single traits is influenced by individuals, who are each represented as an acquired system of cultural traits.

## 1. Introduction

Contemporary cultures differ in many ways from one another, and from cultures existing 100, 1000 or 10 000 years ago. To understand cultural change and the emerging characteristics of cultures, researchers have modelled how cultural information is moulded as it flows from senders to receivers, yielding insights into topics such as social learning [1], cumulative cultural evolution [2–5], the spread of innovations [6], the ebb and flow of fashions [7–10], the influence of different modes of transmission [11–14], cultural adaptation [11,15,16], and the relationship between cultural and genetic evolution (sometimes called gene–culture coevolution [11,17–21]). Most models of cultural transmission focus on how receivers process information obtained from senders. For example, receivers may prefer information that conforms to the majority, evokes strong emotions, or proves useful [2,19,22]. Considerable attention has been paid to how biases [12,19,22,23] or cognitive processes [24–27] in the receiver, often assumed to be innate, can drive cultural evolution in specific directions.

By contrast, less attention has been given to the influence of cultural information on senders and how that impacts transmission. Some researchers suggest that senders, too, can affect the transmission process. For instance, celebrities may have a particularly strong impact on the spread of information. This phenomenon is captured by the concept of prestige bias, where individuals preferentially imitate those they perceive as having high status or prestige, thus contributing to the celebrity’s influence in cultural transmission (e.g. [3,28]). However, the focus of this research has been primarily on how receivers perceive, in this case, the celebrity status of the sender, rather than investigating the factors that contribute to someone becoming a celebrity and how those factors are transmitted. Also, it is often assumed that such biases are preexisting rather than investigated how they can evolve culturally. There are a few exceptions in the literature that consider the dynamics between senders and receivers, such as display filtering of what to transmit versus model filtering of whom to copy [29], which hint at the potential evolutionary implications of this relationship [7,17,30]. These studies emphasize the need to explore both aspects of cultural transmission to better understand the broader mechanisms at play in cultural evolution.

Some examples of how sender traits influence transmission are the ability to make yourself heard in a conversation, give rhetorical arguments, and demonstrate expertise, prestige or trustworthiness. These properties can facilitate transmission of the traits that are exposed through such skills, but the sender traits can also themselves be highly exposed and copied to various degrees. Sender traits that are hard to copy directly, like expertise or skill, can take the form of a goal for the receiver.

Here, we design and use a mathematical model to show that the cultural traits of senders are a major driving force in cultural evolution. Our results indicate that these traits are crucial to maintain cultural transmission in the long term, and for culture to self-organize into elaborate conceptual and material systems. In building our model, we leverage a recent conceptual shift in cultural evolutionary theory, from an early focus on single cultural traits [11,19,31] to modelling multiple traits and their interactions in cultural systems [29,32–36], partly inspired by the theory of complex systems outlined by, for example, Thurner *et al.* [37]. Importantly, we investigate the power of cultural evolutionary processes in the absence of genetic evolution and/or inborn biases. This enables us to study how cultural traits influence each other’s dynamics, without the need for predispositional biases, and to investigate the evolution of trait complexes that endure over time by supporting each other. By doing so, we also stress the importance of modelling individuals as holding multiple traits. This individual–trait duality is necessary for understanding the dynamics of cultural transmission when traits need to be learned in a specific order or have various dependencies on one another.

The structure of this paper is as follows. In §2, we introduce the overarching model employed throughout the study. The dynamics of a singular trait are explored in §3, while §4 delves into the specifics of sender trait dynamics. In §5 we examine the manner in which sender traits influence the evolution of other traits. Finally, we provide a summary and discussion of our key findings in §6.

## 2. General model

The model presented in this paper is a comprehensive model of cultural evolution that traces the evolution of cultural types in a population through both social and individual processes. A cultural type refers to individuals with a particular set of cultural traits. Formally, we consider individuals who can possess or lack an arbitrary number of cultural traits. A cultural type is defined by a specific combination of traits (more details about the definition are provided in the electronic supplementary material). We write *x*_*i*_ for the frequency of type *i*, ${\dot{x}}_{i}$ for its rate of change and track the frequencies of types over time. Single trait frequencies can easily be computed by summing the frequency of types where they are included. We assume random pairwise encounters in a well-mixed population, such that senders of type *i* meet receivers of type *j* in proportion to *x*_*i*_*x*_*j*_. Encounters with senders of type *i* result in receivers of type *j* converting to type *k* at a rate $S_{ijk}$ that may depend on all three types (i.e. the receiver does not necessarily copy all traits of the sender). For example, a sender of type AB interacting with a receiver without traits (type 0) may result in the receiver copying one of the traits, and thus becoming of type A or type B, rather than of type AB like the sender. The sender’s type does not change. We also assume that type *i* can convert to type *k* independent of social interactions, at rate $I_{ik}$. This framework is very general and allows potential transitions between any two types and can be used to model the evolution of incompatible traits, traits that need to be learned in a specific order, or as a result of various combinations of two or more traits. Births and deaths are modelled as individual processes, namely, as conversions of individuals from being of type *i* to being of the naive type 0 (without cultural traits, see also the electronic supplementary material). In the cases, we explore individuals die at the same rate independent of their type. But the model can also be used to study type dependent mortality. These assumptions lead to the following equation for type *k*:

2.1 $${\dot{x}}_{k}=\underset{\text{social processes}}{\underbrace{\sum_{ij}(S_{ijk}x_{i}x_{\;j}-S_{ikj}x_{i}x_{k})}}+\underset{\text{individual processes}}{\underbrace{\sum_{i}(I_{ik}x_{i}-I_{ki}x_{k})}},$$

which is a balance equation taking into account all possible conversions from other types to type *k* (positive terms) and from type *k* to other types (negative terms), see also equation (S1) in the electronic supplementary material. Equations like equation (2.1) have proven useful for describing many complex phenomena [37], such as chemical reaction networks [38] and ecological interactions between species (e.g. the Lotka–Volterra model [39]). All models below have this form, with the coefficients $S_{ijk}$ and $I_{ik}$ set to explore specific phenomena. From the equation, we also derive an *ontogenetic equation* that describes how the frequencies of types change with individual age in a stable cultural environment (see the 'Ontogeny' section in the electronic supplementary material).

We make minimal assumptions to highlight the unique features of cultural evolution and to reduce its complex dynamics to a tractable mathematical model. First, in our models, individuals are born without cultural traits and they can gain and lose traits throughout life. Second, we do not assume any genetic influences on culture, and culture does not affect survival and reproduction. Hence, we focus on the power of cultural evolution alone. The model allows for assumptions on genetic influences to be added (see §6). Since we are claiming that sender traits play a major role in cultural evolution, it is useful to categorize the traits according to how they affect the dynamics of equation (2.1). We make a first distinction between traits that operate during transmission and those that operate apart from transmission. Traits that operate during transmission are further divided depending on whether they operate in the sender or in the receiver of cultural information. It is important to note that a trait can operate both during and apart from transmission, and in both the sender and the receiver.

We thus categorize cultural traits into the following categories.

Social processes operate during transmission:
• Sender traits affect cultural transmission when their bearer serves as the sender of information.
• Receiver traits affect cultural transmission when their bearer receives information.

Individual processes operate apart from transmission:

• Goal traits set targets for individual efforts.
• Production traits produce material or mental results that can be used to achieve goals.

In models of the type developed in this paper, it is always clear what role(s) different traits play. However, making such classifications based on empirical observations may not always be straightforward, because of the diversity of traits and their operation not being transparent. Potential examples of sender traits are speaking with a particular accent, displaying wealth and wearing trendy clothes. Trusting strangers and racial prejudice, on the other hand, are potential receiver traits. In table 1, we suggest groups of traits that can typically be classified into a particular category.

**Table 1.** Examples of trait operation. Social processes operate during transmission, while individual processes operate apart from it. Goals are traits that individuals can strive for, while production traits are those that they have. A trait can operate in several places (e.g. it can be both a sender and a production trait). Social and individual processes are defined formally in equation (2.1).

social processes		individual processes	
sender traits	receiver traits	goals	production traits
communicability	communicability	competencies	practical skills
teaching abilities	learning abilities	products	cognitive skills
sender motivation	receiver motivation	status	knowledge
appearance	sender preferences/biases	wealth	resources
targeted audience		well-being	motivation
what to express	content preferences/biases		training
persuasiveness	openness		
power	response to power		

Apart from transmission, cultural traits can also affect the evolution of other traits through individual learning. For example, one trait might be necessary for the acquisition of another one. Sequence learning has been stressed as important [40]. Whenever a number of traits are necessary for the acquisition of another one, we call the intermediate traits *production traits*. Individual learning does not necessarily need to occur fully sequentially, but a combination of production traits may be necessary for acquiring a specific trait. In this paper, we focus on cases where traits are learned in sequence, but our formal model encompasses the combinatorial case as well.

Below, we use equation (2.1) to track type or trait frequencies over time. We are primarily interested in understanding when a cultural trait can spread and persist, after being introduced in a population at a low frequency. This is similar to mathematical models of genetic evolution that track the fate of initially rare mutations [41]. For brevity, we refer to a rare trait spreading and persisting as the trait ‘evolving’ in the population. Through the main text, we use a simplified indexing of types compared to the electronic supplementary material.

## 3. Sender traits can promote their own transmission

Consider the dynamics of a single sender trait. We have two cultural types: 0 (naive type, without the trait) and 1 (with the trait). Key transmission rates (S in equation (2.1)) for this case are:

— $\;p_{S}\;:=S_{101}$, the rate at which Senders of type 1 convert Receivers of type 0 to type 1;
— $p_{0}\;:=S_{010}$, the rate at which Senders of type 0 convert Receivers of type 1 to type 0.

We also assume a birth–death rate of *β*, and that the trait cannot be gained or lost individually, yielding $I_{01}=0$ and $I_{10}=\beta$ (a more general model is shown in the electronic supplementary material, equation (S2)). Thus the expected lifetime of individuals in this model is 1/*β*. The other elements of S and I can be set to zero (see the electronic supplementary material for a detailed analysis, including the case $I_{01}>0$). With these assumptions, equation (2.1) yields

3.1 $${\dot{x}}_{S}=\;p_{S}x_{S}x_{0}-p_{0}x_{0}x_{S}-\beta x_{S},$$

where $x_{S}$ is the (relative) frequency of individuals with the trait, and $x_{0}=1-x_{S}$ the frequency of individuals without the trait. The first term models transmission of the trait from senders with the trait to individuals without the trait. The second term models trait loss when a sender without the trait influences a receiver to abandon the trait. The last term models trait loss due to death of individuals with the trait. The first two terms are social processes and the last term an individual process.

**Figure 1.**
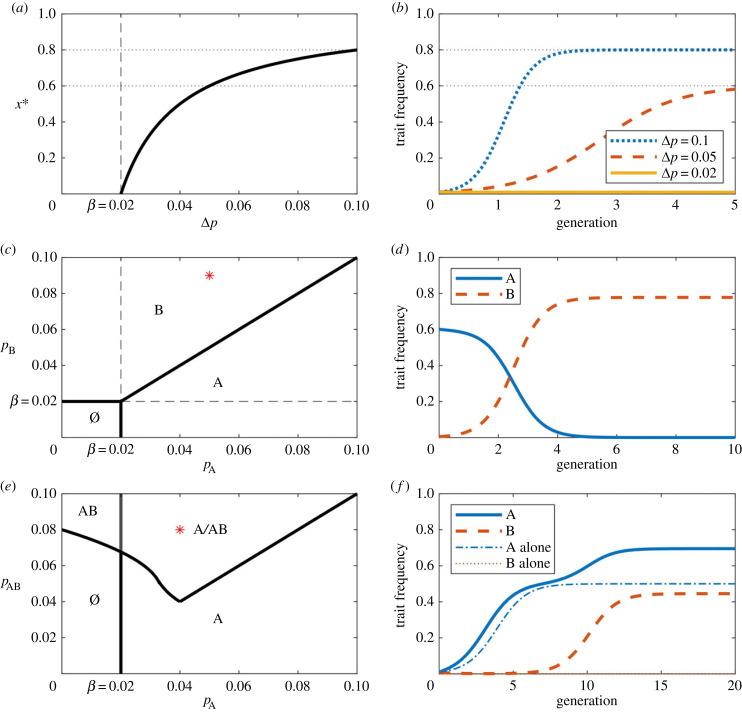
Evolutionary dynamics of sender traits. Panels (*a*,*b*) illustrate the single-trait model in equation (3.1), (*c*,*d*) the competitive sender trait model in equation (4.1) and (*e*,*f*) the complementary sender trait model in equation (4.2). (*a*) Equilibrium frequency of the single-trait model. (*b*) Trajectories for three different values of *p*_S_ − *p*_0_ (initial condition *x*_S_(0) = 0.01) in the single-trait model. The dotted lines indicate the corresponding equilibrium frequencies. (*c*) Phase space in the competitive sender trait model. Letters indicate which trait is stable in which region. (*d*) Trajectory of a new sender trait with higher transmission rate and replacement of the old trait by the new one. The red star in the left panel indicates the parameters used in the right panel, i.e. $\;p_{A}=0.05$ and $\;p_{B}=0.07$, with initial conditions $x_{A}=0.6$ and $x_{B}=0.005$. (*e*) Phase space in the complementary sender trait model. Its properties are discussed in the electronic supplementary material and the condition for the stability of AB are given in electronic supplementary material, Eq. S17. (*f*) Evolution of trait frequencies for the parameters specified by the red star in (*e*). Initial conditions are given by $x_{A}=0$ and $x_{\mathrm{AB}}=0.01$. The birth–death rate *β* = 0.02 for all simulations. The length of one generation is equal to the expected lifetime 1/*β*.

Equation (3.1) has two possible outcomes, illustrated in figure 1*a*,*b*: either the trait disappears, or it stabilizes at frequency

3.2 $${x_{S}}^{⋆}=1-\frac{\beta }{\;p_{S}-p_{0}},$$

(see equation (S7) in the electronic supplementary material for a more general case). The condition for the trait to persist is

3.3 $$\;p_{S}>p_{0}+\beta .$$

In conclusion, sender traits can be under direct cultural selection and can promote their own evolution given that the above condition is fulfilled, that is, given that they increase their own transmission probability sufficiently.

Consider now a similar model for a receiver trait. The key difference is that a receiver trait does not operate when it is transmitted. By definition, it does not influence the efficiency of the sender, and it influences the receiver only after it has been transmitted. Thus, the rate of transmission is the same as for two naive individuals (cf. [17,42]). This leads to a subtle yet consequential change to the dynamics compared with equation (3.1):

3.4 $${\dot{x}}_{R}=p_{0}x_{R}x_{0}-\;p_{R}x_{0}x_{R}-\beta x_{R},$$

where $p_{0}$ ($S_{101}$) is the rate at which receivers without the trait gain the receiver trait R when meeting senders with the trait and *p*_R_ ($S_{010}$) is the rate at which receivers abandon the receiver trait R influenced by senders without that trait. Note the differences between equations (3.1) and (3.4): in the former, the modified rate *p*_S_ applies to the acquisition of the trait, while in the latter, the modified rate *p*_R_ applies to its loss, while the naive rate *p*_0_ operates when acquiring R and losing S. Thus in contrast to a sender trait, a receiver cannot promote its own transmission. See [17,42] for a different derivation of this result using variance statistics, and for additional discussion of differences between sender and receiver traits.

It follows from exploring our equation for a receiver trait in the same way as we explored equation (3.1) that a receiver trait can evolve only if it decreases, rather than increases, the transmission rate sufficiently. In fact, the analogue of equation (3.3) is

3.5 $$\;p_{R}<p_{0}-\beta ,$$

in which case the trait stabilizes at frequency

3.6 $${x_{R}}^{⋆}=1-\frac{\beta }{p_{0}-\;p_{R}}.$$

Given that the above condition is met, it can also be shown that among competing receiver traits, the one with the lowest transmission rate will win [17,42]. Thus, cultural evolution of receiver traits favours decreased cultural transmission. The intuitive reason for this conclusion is that a lower transmission rate protects a receiver trait from being replaced. It also follows from similar reasoning that a cultural trait that does not influence transmission cannot evolve on its own (see electronic supplementary material). However, as we will show below, both receiver traits that promote transmission and transmission of neutral traits can evolve through cultural coevolution with other traits.

## 4. Evolutionary dynamics of sender traits

The results above suggest that sender traits are crucial to cultural evolution, because they can promote cultural transmission while receiver traits tend to oppose it. Here, we explore further how sender traits can drive cultural dynamics by studying models with two sender traits, A and B. We have cultural types 0 (naive), A, B and AB. The social factors array S in equation (2.1) has size 4 × 4 × 4, and the individual factors array I has size 4 × 4. However, we can always set to zero $S_{iij}$ (these terms always cancel out), $S_{ijj}$ and $I_{ii}$ (these terms do not change the receiver’s type). Further simplifications are possible in specific scenarios (see the electronic supplementary material for details).

### (a) Sender traits can compete

Here, we consider mutually exclusive sender traits, such as incompatible ways to influence receivers like having long or short hair, playing a particular musical instrument, or advertising adherence to one political party or another. In this case, we only have three cultural types (0, A and B), because type AB cannot occur. We assume that a type can convert to another by observing it, and that conversions to type *i* occur at rate *p*_*i*_ (*i* ∈ {0, A, B}), with $p_{0}<\;p_{A}<\;p_{B}$. Thus sender trait A transmits more readily than the naive type, and sender trait B more than sender trait A. The resulting equation reads

4.1 $$\left\{ \begin{array}{ccc} {\dot{x}}_{A} & = & \;p_{A}x_{A}(x_{0}+x_{B})-(p_{0}x_{0}+\;p_{B}x_{B})x_{A}-\beta x_{A}\\ {\dot{x}}_{B} & = & \;p_{B}x_{B}(x_{0}+x_{A})-(p_{0}x_{0}+\;p_{A}x_{A})x_{B}-\beta x_{B} \end{array}\right..$$

Analysis of these equations reveals two stable equilibria (see figure 1*c*,*d*, and equation (S10) in the electronic supplementary material). If $\;p_{B}-p_{0}<\beta$, then both sender traits disappear. If $\;p_{B}-p_{0}>\beta$, then trait B persists at non-zero frequency, while trait A disappears. There is no stable equilibrium in which trait A persists. This result extends to any number of mutually exclusive traits (see electronic supplementary material), and implies that cultural evolution selects for the most effective sender traits.

### (b) Sender traits can promote each other

If only one sender trait could persist, then cultures would be exceedingly simple. Here, we begin exploring richer cultural dynamics by considering two sender traits A and B that can support each other’s transmission. As an example, we assume that a supporting trait A can be acquired on its own, while B can be acquired only if the individual already has A. Some specific examples of this are that you need to know how to write before you can learn to write efficiently, you need to learn pedagogical skills (which can be transmitted also by using them) before you can teach them explicitly, or when you have learned who is your targeted audience, then you can also learn how to target your message to that audience. The corresponding dynamical equations (see equation (S11) in the electronic supplementary material) are

4.2 $$\left\{ \begin{array}{ccc} {\dot{x}}_{A} & = & p_{A}x_{A}x_{0}+\;p_{B}x_{\mathrm{AB}}x_{0}-\;p_{B}x_{\mathrm{AB}}x_{A}-\beta x_{A}\\ {\dot{x}}_{\mathrm{AB}} & = & \;p_{B}x_{\mathrm{AB}}x_{A}-\beta x_{\mathrm{AB}}\;\;\;\;\;\;\; \end{array}\right.,$$

where $\;p_{A}$ is the rate at which a naive receiver gains A in encounters with senders that have A, and $\;p_{B}$ is the rate at which receivers without traits gain A or receivers with A gain B in encounters with senders that have B. For simplicity, we assume that traits cannot be lost once acquired. There are three possible outcomes, in which no trait, only A, or both traits A and B become established (see figure 1*e*,*f*, and the electronic supplementary material for a detailed analysis). The persistence of A requires $\;p_{A}>\beta$, and the persistence of both traits requires $\;p_{B}$ to be sufficiently large. Intriguingly, A and B can both get established even when A cannot on its own, that is, if $\;p_{A}<\beta$ and $\;p_{B}$ is large enough.

## 5. Sender traits can drive the evolution of other traits

So far we have considered sender traits that can be copied directly. However, many sender traits, such as physical items like jewellery or clothing, and skills like playing a musical instrument or displaying expertise, cannot be copied directly and require significant individual efforts to be acquired. The following three models explore how sender traits can drive the evolution of other traits, such as goal, production and receiver traits (table 1), and coevolve with them.

### (a) Sender traits can support cultural goals

**Figure 2.**
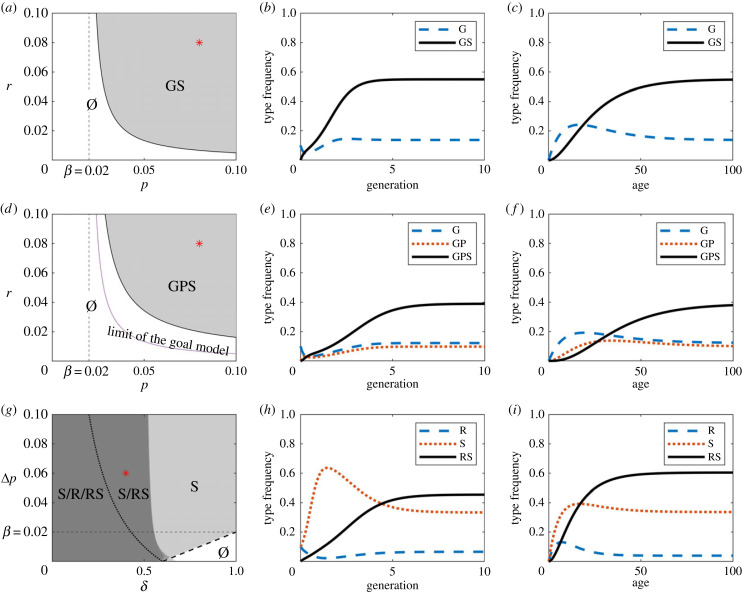
Coevolution of sender traits with other traits. Panels (*a*–*c*) illustrate the goal model in equation (5.1). (*a*) Phase plot of the process. The grey region corresponds to the stability of G and S. (*b*) Dynamics of the system for the parameters corresponding to the red star in (*a*). Initial conditions are given by $x_{G}(0)=0.1$ and $x_{\mathrm{GS}}(0)=0$. (*c*) Ontogeny (individual development) for the parameters corresponding to the red star in (*a*) (for calculation see Ontogeny in the electronic supplementary material). Expected lifetime is 50. Panels (*d*–*f*) illustrate the production–goal model in equation (5.2). (*d*) Stability of G, P and S. For reference, the stability of the goal model is provided as a dotted line for comparison. (*e*) Dynamics of the system for the parameters corresponding to the red star in (*d*). Initial conditions are given by $x_{G}(0)=0.1$ and $x_{\mathrm{GP}}(0)=x_{\mathrm{GPS}}(0)=0$. (*f*) Ontogeny for the parameters corresponding to the red star in (*d*). Panels (*g*–*i*) illustrate the sender–receiver model (equation not shown in the text, see equation (S22) in the electronic supplementary material). (*g*) Phase plot of the process. It also specifies the regions in which a trait is stable on its own (see the electronic supplementary material for details and figures S6 and S7 for phase plots with only R or only S). The dotted line corresponds to the limit of stability for having only R (see electronic supplementary material, figure S6). The phase plot shows the increase of stability of R due to the presence of S. (*h*) Dynamics of the system for the parameters corresponding to the red star in (*g*). Initial conditions are given by $x_{R}=x_{S}=0.1$, and $x_{\mathrm{RS}}=0$. (*i*) Ontogeny for the parameters corresponding to the red star in (*g*).

Here, we show that a sender trait S can drive the evolution of another trait and coevolve with it. When a sender trait S cannot be copied directly, we assume that observing S transmits to receivers a ‘goal’ trait G that can later result in the receiver acquiring S through individual efforts. For example, a sender with a trendy jacket may result in a receiver acquiring the goal or preference for the jacket and then buying or making a similar jacket through individual efforts with some delay. G can also be viewed as a preference for S or some other motivation to obtain S [7,9]. We assume that S can be acquired only by individuals with G, that G does not enhance transmission by itself (since it is not a sender trait), and that only senders with S can transmit G to naive receivers. We also assume that once G is obtained, S can be acquired through individual efforts. Thus the two traits support each other since G is needed for the acquisition of S and S promotes the social transmission of G. These assumptions give rise to two types of individuals (in addition to the naive type), one with just the goal G and one with both the goal and the sender trait GS, and yield the following dynamics (see equation (S18) in the electronic supplementary material):

5.1 $$\left\{ \begin{array}{ccc} {\dot{x}}_{G} & = & px_{\mathrm{GS}}x_{0}-(r+\beta )x_{G}\\ {\dot{x}}_{\mathrm{GS}} & = & rx_{G}-\beta x_{\mathrm{GS}}\;\;\;\; \end{array}\right.,$$

where *p* is rate whereby GS senders transmit G to naive individuals, *r* the rate at which G individuals acquire S through individual efforts, and *β* the birth–death rate. This system has a unique solution with *x*_G_ and GS greater than zero provided *p* > *β*(1 − *β*/*r*) (see equation (S19) in the electronic supplementary material). This condition can always be satisfied for large enough *p*, that is, if S enhances transmission sufficiently. However, if *r* is small (S is difficult to acquire), then the population will consist primarily of individuals with G but without S. As *r* increases, the share of the population with both the goal and the trait also increases (see electronic supplementary material, figure S3, first panel). The model is illustrated in figure 2*a*–*c*. Figure 2*a* is a phase plot showing for which parameter combinations GS can evolve and be maintained. Figure 2*b* shows an example of evolution of the G and GS types from low frequencies. The parameters used are indicated with a red dot in the phase plot. In these settings, it takes a few generations to converge to a stationary state in which about 55% of the population are of type GS and have the two traits, while about 15% have only the goal trait G. Figure 2*c* illustrates the ontogeny of types at equilibrium for the same parameters. The ontogeny shows that individuals acquire the goal G first and then rapidly acquire the sender trait S, thus becoming of type GS. Note that the evolution and the ontogeny do not exactly resemble each other.

### (b) Sender traits can support production traits

We can broaden the interpretation of G to anything that may be necessary for acquiring a sender trait S, such as a mental or behavioural skill, money, or a physical item such as a tool. In fact, acquiring a sender trait S may require many such traits. For example, persuasive speaking may be leveraged by pronunciation, vocabulary, metaphors and soon, and hearing a catchy tune and wanting to reproduce it means you need to practice. We call *production traits* those traits that, in addition to goals, are necessary to acquire a sender trait. Here, we show that a sender trait can support the simultaneous evolution of production and goal traits. We illustrate this phenomenon by adding a single production trait to the previous model, but one may also consider production chains (traits that have to be acquired in a strict sequence) and production networks with more complex dependencies between traits.

We assume that possessing the goal trait G makes it possible to acquire first a production trait P and then a sender trait S. The possible cultural types are G, GP, GPS and the naive type. We assume that G is learned socially, and P and S individually. For simplicity, we assume that P and S cannot be acquired without G, and that G cannot by transmitted without S (see the electronic supplementary material for a model in which G and GP individuals also contribute to social learning). These assumptions lead to

5.2 $$\left\{ \begin{array}{ccc} {\dot{x}}_{G} & = & px_{\mathrm{GPS}}x_{0}-(r+\beta )x_{G}\;\\ {\dot{x}}_{\mathrm{GP}} & = & rx_{G}-(r+\beta )x_{\mathrm{GP}}\;\\ {\dot{x}}_{\mathrm{GPS}} & = & rx_{\mathrm{GP}}-\beta x_{\mathrm{GPS}}\;\;\;\;\; \end{array}\right.,$$

where we have assumed common rates *p* and *r* for acquiring any trait socially or individually, respectively, and *β* for the birth–death rate (a more general model is treated in the electronic supplementary material, equation (S20)). This system has a unique solution with *x*_G_, GP and GPS greater than zero provided *p* > *β*(1 − *β*/*r*)^2^ (see equation (S21) in the electronic supplementary material). This condition can always be satisfied for large enough *p*, that is, if S enhances transmission sufficiently. However, if *r* is small (S and P are difficult to acquire), then the population will consist primarily of individuals with G but without S or P. As *r* increases, the share of the population with all three traits also increases (see electronic supplementary material, figure S5). This is similar to the goal model in equation (5.1). Note that it is straightforward to increase the number of intermediate production traits and the results generalize easily. The model is illustrated in figure 2*d*–*f*. Figure 2*d* is a phase plot describing for which parameter combinations GPS can evolve and be maintained. Figure 2*e* shows an example of evolution, and figure 2*f* the ontogeny of types at equilibrium for the same parameters. The evolution shows that it takes about five generations to reach equilibrium frequencies and the ontogeny shows that individuals acquire the goal G, the production trait P and the sender trait S in sequence. Recall that we are plotting type frequencies and that trait frequencies can be recovered by adding the types containing a specific trait.

### (c) Sender traits can support receiver traits

Here, we show that a sender trait S can support the evolution of a receiver trait R that would otherwise disappear from the population. We assume that transitions between cultural types depend jointly on whether the sender has S and the receiver has R. There are thus four transition rates:

$p_{0}$: the sender lacks S and the receiver lacks R.
$p_{R}$: the sender lacks S and the receiver has R.
$p_{S}$: the sender has S and the receiver lacks R.
$p_{\mathrm{RS}}$: the sender has S and the receiver has R.

For simplicity, we assume that $\;p_{R}=\;p_{S}=p_{0}+\Delta p$ and $p_{\mathrm{RS}}=p_{0}+2\Delta p$. With reference to the model in §3 (to which the present model reduces when the population has only one of R and S), this means that the receiver trait cannot be sustained on its own in the population, while the sender trait can. To explore a wider range of possible dynamics, we also consider the condition that the absence of a trait does not transmit as readily as its presence (typically, the absence of a trait is less visible than its presence, e.g. you would more often note that someone is wearing a red jacket than noticing that someone is *not* wearing a blue or a green jacket), by multiplying the transmission by a factor *δ* ≤ 1. For example, we assume conversions from R to 0 to occur at rate $\delta p_{0}$.

The formal dynamical system that these assumptions imply is not very informative in itself and is provided in the electronic supplementary material for completeness (equation (S22)). Reducing *δ* below a given threshold leads to R evolving on its own, since this leads to an effective reduction of the rate of unlearning (see §3, and the electronic supplementary material, figure S6, for a phase plot for R). The stability condition for S also changes, and below a certain threshold, S becomes unconditionally stable. Figure 2*g*–*i* illustrate this model. Figure 2*g* shows the phase plot. The dotted line represents the limit of stability of R as the only trait and the thick dashed line that of S. Note that there is an intermediate white region where R is present exclusively along with S. This shows that the stability domain of R is extended by the presence of S, and thus that there are situations where R can exist only aided by S. Figure 2*h* shows an example of evolution, and figure 2*i* the ontogeny of types at equilibrium for the same parameters. In this model, the difference between evolution and ontogeny is more pronounced than in the other two cases in the figure. In evolution, the lead of the S type is more pronounced. Note also that in the ontogeny, the R type first increases and then decreases with age since individuals with R acquire the sender trait S and become of type RS.

## 6. Discussion

A successful theory of cultural evolution must be capable of explaining the emergence and transmission of complex cultural phenomena, including pottery and other crafts, piano play, belief systems, mental arithmetic and literacy. In this paper, we have presented a comprehensive model of cultural evolution that traces the evolution of cultural types in a population driven by both social and individual processes (see equation (2.1)). Our model identifies trait interactions both among and within individuals and thus effectively addresses the trait–individual duality by modelling how initially naive individuals develop by successively acquiring cultural traits. Our analyses also recognize the importance of representing the individual as a trait complex, that is, as possessing a system of cultural traits, and builds on recent attempts at modelling multiple traits and their interactions in cultural systems [29,32–36].

Our results suggest that cultural traits that operate in senders constitute a major force in cultural evolution since they can promote their own transmission as well as that of other traits, such as receiver (electronic supplementary material, equation (S22)), goal (equation (5.1)) and production (equation (5.2)) traits, that support the establishment of sender traits. Our investigations focus on sender traits and do not contain any complete analysis of receiver traits, whose evolutionary dynamics is different [17]. In fact, the evolutionary pressure acting on receiver traits concerns traits already in the receiver rather than on traits being transmitted. Future work should investigate the extent to which receiver traits can also be direct drivers of cultural evolution, for example through the coevolution of two receiver traits mutually supporting each other. Also, future work could go beyond our model assumption of well-mixed populations. Structured interaction in networks might amplify or mitigate the effects we have found here and the formation of social ties is itself part of the sender–receiver dynamics.

A consequence of our analyses is that we should expect individuals to strive to become cultural role models by investing time and effort in acquiring skills, knowledge, and resources. This effort transforms them into effective transmitters of cultural traits. In reality, individuals become efficient senders only after acquiring a large number of traits. Our small-scale examples of purely cultural evolution raise an important question for further investigation: what can cumulative cultural evolution achieve on its own, with sender traits and social transmission as the main driving force? Biological evolution has created sophisticated organisms with complex organs such as eyes, immune and nervous systems, lungs and flight capabilities; all of this has evolved with natural selection as the main driving force. It seems plausible that a slightly different driving force, as described in this paper, could be what enables cultural evolution to give rise to a similar multitude of complex and fantastic phenomena, both material and mental, such as bicycles, chemistry, religions and operas.

A comparison between cultural evolution and signal evolution in biological evolution is relevant here. Communication systems in biological organisms evolve driven by common and conflicting interests among senders and receivers and have resulted in many spectacular phenomena [43,44]. In sexual selection, this has given rise to spectacular sexual displays that combine informative and purely aesthetic elements [45]. The cultural evolution of sender traits is subject to cultural selection which shares similarities with the biological evolution of signals.

A common objection to the power of cultural evolution working on its own is that social transmission seems too imprecise compared to the transmission of genetic information from parents to children [26,46,47]. Meanwhile, we also know that many complex cultural phenomena are accurately transmitted between generations and mastered by most individuals, such as reading and writing, basic arithmetic and car driving. Many of these skills are of very recent origin, ruling out any inborn guidance. To explain these phenomena, we need a broader view of cultural transmission that includes incremental processes and combinations of social learning and individual training [26,40,48]. Already rather simple skills like tying shoe laces depend on both social and individual processes [49]. Adults provide the goal of producing a knot, and instructions on how to do that, but individual efforts are also required for the child to master this skill. The fact that both practical and cognitive skills and knowledge are acquired through incremental processes, guided by parents, other adults, and peers, is a general observation in developmental psychology and related fields such as educational science and linguistics. In several of our models, namely the goal (equation (5.1)), the production-goal (equation (5.2)) and the complementary sender traits (equation (4.2)) model, the individuals develop by acquiring the traits incrementally. Our general model can be used to study incremental acquisition, and thereby social transmission of complex cultural phenomena, by combining individual and social processes, where the latter can incorporate guidance from others who provide increasingly challenging objectives and skills.

Cumulative cultural evolution also requires generation of new cultural phenomena. It is common to view innovation processes as different from transmission processes, but they have many similarities. Our general model can account for innovation processes through individual, and sometimes social, processes, when individuals produce innovations together. Individual efforts, trying to fulfil inborn or cultural goals, can sometimes give rise to new skills, and cultural goals can be modified or invented to facilitate the innovation of new skills. It is the interaction between the innovation of goals and skills that makes cultural evolution powerful, given sufficient plasticity in both cases. There are several possible scenarios to explore when it comes to the structure of innovations, and how new traits are generated based on existing traits. For example, new traits can emerge from modification, differentiation or combination of existing traits [35,50–52]. The models discussed in this paper only address the initial stages of such cumulative evolution. However, they offer insights into how the development of a more intricate culture can be explicitly modelled through feedback loops that emerge through interactions among sender, receiver, goal and production traits (see §5).

Most current theories of cultural evolution are based on the premise that innate biases, social learning strategies and other inherent factors play a role in directing cultural evolution towards productive outcomes [1,12,19,26,46]. However, it is unclear whether such inborn cultural capacities and goals can account for all the complexity of human culture, including many cultural phenomena of very recent origin, for which biological evolution is too slow. An alternative explanation is that both goals and cultural capacities, including cognitive mechanisms for cultural learning and thinking, are subject to cultural evolution, giving cultural evolution more opportunities for self-organization [5,25,40,53].

Understanding how interacting entities can self-organize and give rise to complex phenomena is studied in complex systems theory [37,54,55] and has been applied to study, for instance, chemical reaction networks, the origin of life, and how species interactions form ecosystems [37–39,55]. Our general model applies the theory of complex systems to cultural evolution. In this paper, we show that our model has the potential of generating complex culture driven by cultural evolution of sender traits, with minimal assumptions on inborn support for cultural evolution. In particular, both the goal model (5.1) and the production–goal model (5.2) contain feedback loops characteristic of complex systems. It is worth noting that our model can include a wide range of assumptions, ranging from inborn mechanisms and biases, to various innovation and developmental processes. Future theoretical research should investigate more scenarios of trait interactions, systems, self-organization and cumulative evolution. It should explore the scope of cultural evolution in terms of cognitive abilities, cultural goals and biases, and try to sort out the role of inborn factors in cultural evolution, what they are, and how they operate.

## Data Availability

The data are provided in electronic supplementary material [56].
